# The Role of Pref-1 during Adipogenic Differentiation: An Overview of Suggested Mechanisms

**DOI:** 10.3390/ijms21114104

**Published:** 2020-06-09

**Authors:** Carina da Silva, Chrisna Durandt, Karlien Kallmeyer, Melvin A. Ambele, Michael S. Pepper

**Affiliations:** 1 Institute for Cellular and Molecular Medicine, Department of Immunology, and SAMRC Extramural Unit for Stem Cell Research and Therapy, Faculty of Health Sciences, University of Pretoria, PO Box 2034, Pretoria 0001, South Africa; carinacrdasilva@gmail.com (C.d.S.); chrisna.durandt@up.ac.za (C.D.); karlienkallmeyer@gmail.com (K.K.); melvin.ambele@up.ac.za (M.A.A.); 2 Department of Oral Pathology and Oral Biology, School of Dentistry, Faculty of health Sciences, University of Pretoria, PO Box 1266, Pretoria 0001, South Africa

**Keywords:** adipogenesis, Pref-1, adipose-derived stromal/stem cells, Wharton’s jelly derived stromal/stem cells, transcription factor, differentiation, Notch signaling, MAPK kinase signaling

## Abstract

Obesity contributes significantly to the global health burden. A better understanding of adipogenesis, the process of fat formation, may lead to the discovery of novel treatment strategies. However, it is of concern that the regulation of adipocyte differentiation has predominantly been studied using the murine 3T3-L1 preadipocyte cell line and murine experimental animal models. Translation of these findings to the human setting requires confirmation using experimental models of human origin. The ability of mesenchymal stromal/stem cells (MSCs) to differentiate into adipocytes is an attractive model to study adipogenesis in vitro. Differences in the ability of MSCs isolated from different sources to undergo adipogenic differentiation, may be useful in investigating elements responsible for regulating adipogenic differentiation potential. Genes involved may be divided into three broad categories: early, intermediate and late-stage regulators. Preadipocyte factor-1 (Pref-1) is an early negative regulator of adipogenic differentiation. In this review, we briefly discuss the adipogenic differentiation potential of MSCs derived from two different sources, namely adipose-derived stromal/stem cells (ASCs) and Wharton’s Jelly derived stromal/stem cells (WJSCs). We then discuss the function and suggested mechanisms of action of Pref-1 in regulating adipogenesis, as well as current findings regarding Pref-1’s role in human adipogenesis.

## 1. Introduction

Over one third of the world’s population is overweight or obese [1,2]. Based on current trends, estimations predict that 60% of the global population will be obese or overweight by 2030 [1,2]. This is a huge concern, as obesity is a major risk factor for several comorbidities such as diabetes mellitus, hypertension, cardiovascular disease and cancer [3,4,5]. The strong association of obesity with chronic comorbidities contributes significantly to overburdened health care systems globally [6,7]. The obvious need for novel interventions to combat obesity has resulted in an active interest in identifying novel therapeutic targets to manage obesity and treat obesity-associated metabolic disorders.

Identifying novel therapeutic targets requires a good understanding of the regulatory elements involved during adipogenesis (fat cell formation). It is; however, of concern that the regulation of adipocyte differentiation has predominantly been studied using the murine 3T3-L1 preadipocyte cell line and murine experimental animal models. Translation of these findings to the human setting is uncertain and requires confirmation using experimental models of human origin.

Mesenchymal stromal/stem cells (MSCs) have the ability to differentiate into various mesodermal cell lineages, such as osteoblasts, myoblasts and adipocytes [8,9]. The ability of MSCs to differentiate into adipocytes is an attractive model to study adipogenesis in vitro. Furthermore, differences in the ability of MSCs isolated from different sources to undergo adipogenic differentiation, may be useful in investigating the different elements responsible for this process. In this review, we briefly discuss the different types of adipose tissue and the ability of MSCs to differentiate into adipocytes, focusing on adipose-derived stromal/stem cells (ASCs) and Wharton’s jelly derived stromal/stem cells (WJSCs). The function and suggested mechanisms of action of Pref-1 in regulating adipogenesis, as well as current findings regarding Pref-1′s role in human adipogenesis, is then reviewed.

## 2. Adipose Tissue

**Figure 1:**
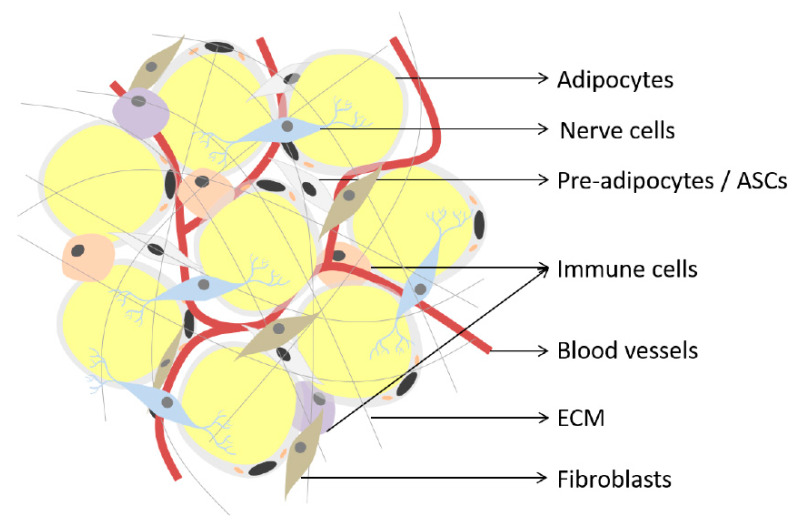
Schematic representation of adipose tissue. Adipose tissue is comprised of adipocytes, preadipocytes, adipose-derived stromal/stem cells (ASCs), immune cells, nerve cells, fibroblasts, blood vessels and extracellular matrix (ECM), among others. Image created by C.d.S.

Adipose tissue is an endocrine organ comprised of terminally differentiated adipocytes, preadipocytes, ASCs, fibroblasts, endothelial cells, nerve cells and immune cells, which function together to maintain homeostasis within the tissue (Figure 1) [10,11]. There are two main types of adipose tissue in mammals: white and brown adipose tissue [12,13,14], which differ regarding their origin, function and morphology.

White adipose tissue (WAT) is found throughout the body [12,13,15], and is mainly involved in energy storage. WAT is further subdivided into visceral and subcutaneous adipose tissue. Visceral WAT is located mainly around organs, while subcutaneous WAT is located directly under the skin [13]. Brown adipose tissue (BAT) is involved in energy expenditure and thermogenesis in infants, and is located at specific sites including the upper back (interscapular region) and around the kidneys (perirenal) [13,16]. A unique feature of brown adipocytes is the expression of uncoupling protein 1 (UCP1), a mitochondrial protein implicated in energy expenditure [16,17]. During cold exposure, the sympathetic nervous system is stimulated and norepinephrine is released which results in BAT producing heat through non-shivering thermogenesis [12]. To do so, fatty acids are released through lipolysis and used by UCP1 for heat production [13,18].

A second brown adipocyte type, known as beige adipocytes, has also been described [16,19,20,21]. Several factors induce the differentiation of precursors, located within WAT depots, into beige adipocytes. Beige adipocytes are also referred to as brite or brown-in-white adipocytes. Factors that induce beige adipocyte differentiation include chronic cold acclimatization, exercise, and long-term treatment with proliferator-activated receptor gamma (PPARγ) agonists, among others [16,22].

**Figure 2:**
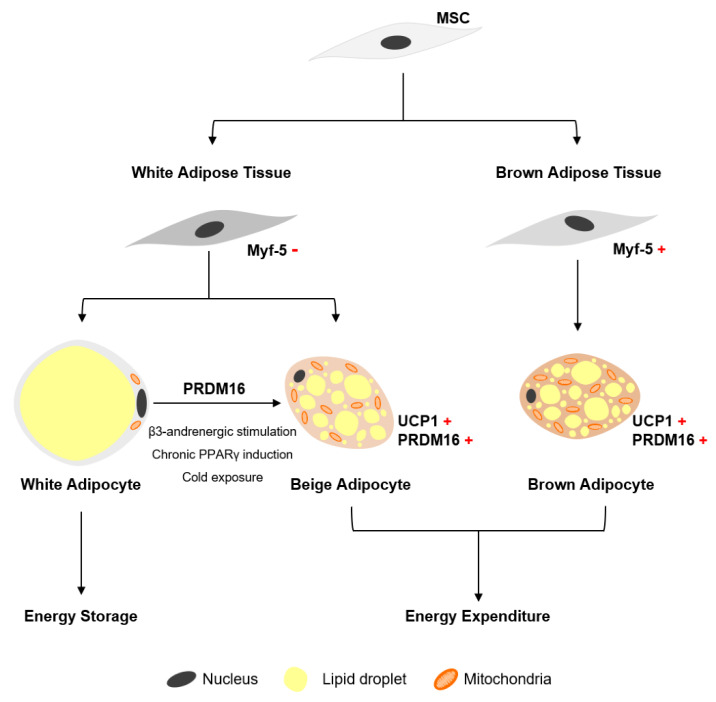
Origin of adipocytes. White and beige adipocytes arise from different precursor cells in their respective adipose tissue depots. White adipocytes are involved in energy storage and contain one unilocular lipid droplet which occupies most of the cytosol. Beige and brown adipocytes are involved in energy expenditure through UCP1. MSC, mesenchymal stromal/stem cell; Myf-5, myogenic factor 5; PRDM16, PR domain containing 16; UCP1, uncoupling protein 1 Image adapted by C.d.S. from Sarjeant and Stephens et al. (2012) [12].

Mature white adipocytes are large, roundish cells which are characterized by a single large lipid droplet which occupies most of the cytosol and contains few mitochondria (Figure 2) [19]. Energy is stored in the form of triglycerides within the unilocular lipid droplet [16]. Brown adipocytes are more ellipsoid in shape, mitochondria rich and contain multiple lipid droplets within the cytosol (Figure 2) [12]. Beige adipocytes are roundish cells that, similar to brown adipocytes, are UCP-positive, mitochondria rich and contain multiple lipid droplets [13,16,23].

White adipocytes originate from MSCs within adipose tissue, also known as ASCs, that do not express myogenic factor 5 (Myf-5) (Figure 2) [12,13]. In contrast, brown adipocytes arise from Myf-5-expressing (Myf-5-positve) myotomal precursors (Figure 2) [16,19]. Beige adipocytes arise from a unique set of precursors, still to be defined, that reside within WAT depots [24]. Wang and colleagues (2013) neatly demonstrated, using a pulse-chase fate-mapping technique, that beige adipocytes are not the product of trans-differentiation of pre-existing white adipocytes in WAT depots [25]. The origin of adipocytes has not been fully elucidated, and several other cells, such as vascular endothelial cells and neural crest cells, have been implicated as possible precursors of both brown and white adipocytes [19].

## 3. Mesenchymal Stromal/Stem Cells

MSCs are a heterogeneous population containing a subset of multipotent adult stem cells that were first discovered by Friedenstein and colleagues in murine bone marrow [26,27,28], referred to by the authors as bone marrow-derived MSCs (BM-MSCs). Since their initial discovery, MSCs have been found to be present in most human adult tissues where they play an important role in cell replacement and regeneration, secretion of bioactive molecules and immune regulation [26,29]. The adipogenic, osteogenic and chondrogenic differentiation capacity of multipotent MSCs is well described [28,30], but studies suggest that MSCs also have the ability to differentiate into other cell types such as myocytes and neural cells, albeit that this is not yet well described/accepted [28,29].

Bone marrow is a common source of MSCs; however, bone marrow aspiration is invasive and contains a small number of MSCs (0.001–0.01% of total nucleated cells) [31]. Furthermore, the number of MSCs present in bone marrow declines with age [26,32]. These factors make bone marrow a less attractive source for isolating MSCs for cell therapy applications. On the other hand, adipose tissue obtained from liposuction and abdominoplasty procedures and the post-natal umbilical cord (UC), are generally considered biological waste, and their use is consequently associated with minimal ethical concerns [33,34,35]. This, has resulted in adipose tissue and UC gaining popularity as sources of MSCs [36,37,38]. The reported MSC yields from bone marrow and UC are highly variable, but seem to be comparable [39]. However, the efficiency of isolating MSCs from adipose tissue is significantly greater [39].

The high variability in yields when MSCs are isolated from UC or placenta potentially results from the anatomical complexity of these tissues. MSCs can be isolated from various anatomical regions of the UC, including the amniotic epithelial membrane, the sub-amnion cord lining, intervascular gelatinous connective tissue, known as Wharton’s jelly (WJ), and the perivascular region surrounding the umbilical blood vessels [40,41,42]. MSCs can also be isolated from the fetal side of placenta, the cord-placenta junction or the maternal side of placenta [40]. The yield and the differences in MSC properties may thus be due to differences in the anatomical regions from which these cells are isolated.

### Adipogenic Differentiation Capacity of hASCs and hWJSCs

Adipogenic differentiation is one of the criteria that need to be fulfilled when characterizing MSCs isolated from different tissue sources [43]. Consequently, a large number of studies have compared the adipogenic differentiation potential of MSCs isolated from different tissues. It is clear from these studies that the differentiation characteristics of MSCs differ based on the tissue source or the anatomical site from which the MSCs are isolated [30,44]. The adipogenic differentiation potential of MSCs isolated from different human-derived tissue sources is summarized in Table 1. We have limited ourselves to those studies that either use ASCs or BM-MSCs as reference cultures. As mentioned previously, ASCs are known for their superior adipogenic differentiation potential, while BM-MSCs were the first MSC cell type to be identified/isolated, and are thus often used as a reference standard in MSC studies.

**Figure 3:**
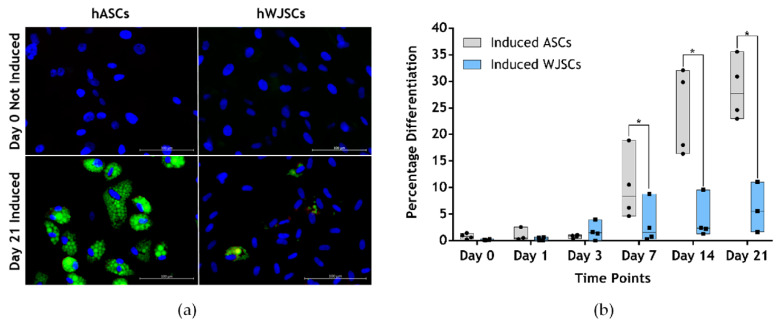
Adipogenic differentiation potential of hASCs and hWJSCs. (**a**) Microscopy images of Day 0 (prior to induction) and Day 21 induced hASCs and hWJSCs. Cells were stained with a nuclear dye Vybrant^®^ DyeCycle Violet (blue) and a lipophilic dye Nile Red (green). Scale bars: 100 μm. Magnification: 20×. (**b**) The percentage of hASCs and hWJSCs that differentiated into adipocytes was determined via a flow cytometric Nile Red assay [62]. Each dot or square within the floating bars represents an independent hASC and hWJSC culture. Four cultures of each were included in the study. The horizontal lines within the bars represent the population median. Statistical significance between the two cell types at the various time points is displayed with an asterisk when * *p* < 0.05 C. hASCS and hWJSCs displayed the following phenotype: CD36+/CD44+/CD45-/CD73+/CD90+/CD105+ (not shown). Unpublished, original data.

Very few studies have directly compared the adipogenic differentiation potential of human ASCs (hASCs) and human WJSCs (hWJSCs) in the same study (Table 1). Ragni and colleagues (2013) [45] and Amable and colleagues (2014) [46] showed that hASCs displayed enhanced adipogenic differentiation when compared to hWJSCs, while Hu and colleagues (2013) [47] observed no difference between these two cell types. Our group has observed that hWJSCs differentiate poorly into adipocytes. On average, 2.91% ± 2.72% of hWJSCs (*n* = 3 independent primary cultures) differentiated into adipocytes after a 21-day adipogenic induction period, compared to 28.50% ± 2.91% (*n* = 3) in the case of hASCs (Figure 3a,b). It is clear from our observation and the studies summarized in Table 1 that although hASCs and hWJSCs display similar morphological and phenotypic characteristics [47,48], they differ with respect to their adipogenic differentiation potential. hASCs and hWJSCs also differ with respect to their proliferation rates (hWJSCs have a higher proliferation rate) and cytokine secretion profiles [47].

The reasons behind the differences observed in differentiation potential between hASCs and hWJSCs are not known, and require further investigation. However, the differences could be exploited as an in vitro model to understand the molecular regulators of adipogenesis. It is likely that several factors determine the ability of MSCs to differentiate into a specific cell type. Pierdomenico and colleagues (2011) suggested that the physiological environment of MSCs affects their differentiation capabilities [63]. These investigators reported that hWJSCs, which were isolated from umbilical cord collected from infants of diabetic mothers, displayed improved adipogenic differentiation capacity compared to hWJSCs isolated from umbilical cord obtained from infants of non-diabetic donors [63]. Xu and colleagues (2017) suggested that MSC fate is controlled by the methylation status of transcription factor genes, and that epigenetic memory plays a role in the differential differentiation capacities of MSCs derived from different sources [30].

## 4. Adipogenesis

Adipogenesis is a complex, multi-step process in which precursor cells differentiate into either mature brown or white adipocytes [12,15,46]. Studies using the 3T3-L1 cell line have shown that white adipogenesis consists of several phases including (i) cell commitment; (ii) mitotic clonal expansion; and (iii) terminal differentiation [64,65]. The stages in humans are less well defined.

**Figure 4:**
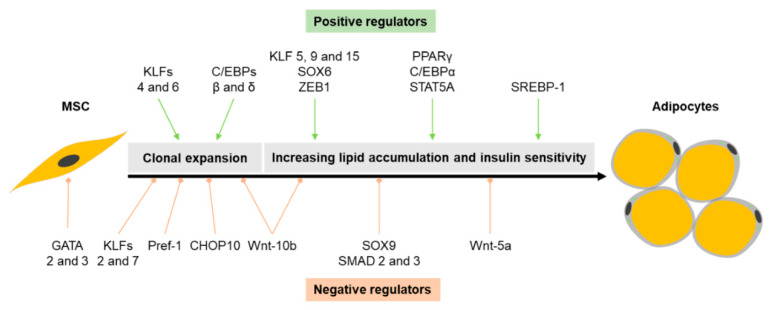
Positive and negative regulators of white adipogenesis. Adipogenesis is tightly regulated by several transcription factors that are expressed at different stages during the differentiation pathway. Image adapted (with permission) by C.d.S., K.K. and M.A. from Sarjeant and Stephens et al. (2012) [12]. MSC, Mesenchymal stromal/stem cell; AP-1, activating protein-1; KLFs, Krüppel-like factors; C/EBP, CAAT-enhancer binding proteins; PPARγ, Peroxisome proliferator-activated receptor gamma; STAT, Signal transducer and activator of transcription; SREBP-1, sterol regulatory element binding protein 1; Pref-1, preadipocyte factor 1; Wnt, Wingless/Integrated protein; SOX 6 and 9; Sex-Determining Region Y-Box 6 and 9; SMAD 2 and 3, Mothers against decapentaplegic homolog 2 and 3; CHOP10, C/EBP homologous protein 10; ZEB1, Zinc finger E-box-binding homeobox 1.

During the cell commitment stage, MSCs commit to undergo differentiation into preadipocytes [64]. Murine preadipocytes then undergo two rounds of mitosis during mitotic clonal expansion [65], which is an important step as the unwinding of DNA allows transcription factors to bind and initiate a well-controlled cascade required for terminal white adipogenic differentiation (summarized in Figure 4) [66,67,68]. Brown/beige adipocyte differentiation and activation is also regulated by sequential activation of a series of transcription factors specific to each of these adipocyte types. However, several different pathways, depending on the stimulus received, may be involved in brown and beige adipogenic differentiation [24,69].

### 4.1. Transcriptional Regulation of White Adipogenesis

Murine preadipocytes express high levels of preadipocyte factor-1 (Pref-1), CCAAT/enhancer binding protein (C/EBP) homologous protein (CHOP) and GATA transcription factor (Figure 4). Elevated levels of these molecules as well other negative regulators of adipogenesis such as sex Determining Region Y-Box 9 (SOX 9) and Mothers against decapentaplegic homolog 2 and 3 (SMAD 2 and 3) suppress adipogenic differentiation, hence the requirement for down-regulation of these molecules in order for adipogenic differentiation to proceed [12,70]. Pref-1 is one of the key negative regulators of adipogenesis [71,72,73,74]. C/EBPα and PPARγ are the main transcription factors that regulate adipogenesis [67,68], with PPARγ being the defining master regulator [12,13,67,68,75,76]. Up-regulation of C/EBPα and PPARγ ends the mitotic clonal expansion phase and leads to terminal differentiation [70]. During terminal differentiation, end-stage adipogenesis-associated genes are up-regulated, allowing preadipocytes to acquire all the morphological, genetic and biochemical characteristics of a mature adipocyte. These features include the up-regulation of enzymes responsible for fatty acid biosynthesis and transport, as well as triglyceride synthesis.

In vitro adipogenic differentiation of MSCs is induced by exposing the cells to a cocktail of compounds consisting of dexamethasone (DXM), 3-isobutyl-1-methylxantine (IBMX), insulin and fetal bovine serum (FBS). Insulin acts through the insulin-like growth factor 1 (IGF-1) receptor to initiate mitotic clonal expansion and also plays a role in promoting lipid accumulation [13,64]. FBS down-regulates the early transcription factor CHOP10, releasing C/EBPβ, which in turn activates C/EBPα and PPARγ [77]. IBMX, a phosphodiesterase inhibitor, activates the cAMP-dependent protein kinase pathway and up-regulates Pref-1, C/EBPβ and C/EBPδ [78]. The synthetic glucocorticoid, DXM, up-regulates C/EBPβ and PPARγ, while down-regulating Pref-1 [13,70,72]. Once activated, PPARγ binds to and activates the promoters of adipocyte specific genes such as fatty acid binding protein 4 [FABP4, also known as adipocyte protein 2 (aP2), an adipocyte selective fatty acid binding protein], and phosphoenolpyruvate carboxylase (PEPCK, which is involved in gluconeogenesis) [79]. Other positive regulators of adipogenesis include sterol regulatory element binding protein-1 (SREBP-1), signal transducer and activator of transcription (STAT) transcription factor 3 and STAT 5A, SOX6, ZEB1, among others [12,13,79].

### 4.2. Transcriptional Regulation of Brown Adipogenesis

There are several genes that distinguish brown adipocytes from white adipocytes (reviewed in [24,69]). In addition to *Ucp1*, some other brown adipocyte-associated genes include *Ppara* (fatty acid metabolism), *Cidea* (lipid droplet remodelling), *Dio2* (thyroid hormone metabolism), *Elovl3* (fatty acid elongase), *Pgc1α* (mitochondrial biogenesis) and *Cpt1b* (fatty acid transport) [24]. Central to the regulation of different pathways, such as brown adipocyte differentiation and adaptive thermogenesis, is the PR domain zinc finger 16 (Prdm16)/Cebpβ complex. PRDM16 is not expressed by white adipocytes and plays an essential role in regulating brown adipogenic differentiation [24,80,81].

Under the control of the sympathetic nervous system (SNS), cold exposure induces Myf5+ mesodermal precursors or beige/brown adipocyte precursors residing in WAT depots to differentiate into beige brown adipocytes [24,80,82,83,84]. Adrenergic signals, such as noradrenaline and catecholamines, are released, which bind to β-adrenergic receptors to activate the downstream differentiation pathways [82,85,86]. Brown and beige adipogenic differentiation requires the up-regulation of early transcription factors, such as early B-cell factor-2 (EBF2) [87], PPARγ coactivator 1A (PGC1α) [88], nuclear factor I-A (NFIA) [89] and others. All of these transcription factors play a role in the up-regulation of adipogenesis-associated genes such as *C/EBPβ* and *PPARγ*. The brown adipogenesis master regulator, PRDM16, binds to the active form of C/EBPβ [90] to regulate several processes, such as browning of WAT, fatty acid oxidation and mitochondrial metabolism [69]. Foxhead P1 (Foxp1) acts as a negative regulator of brown/beige adipogenic differentiation and thermogenesis by preventing the PRDM16/CEBPβ complex from up-regulating PPARγ and β3-AR expression [82].

## 5. Preadipocyte Factor 1 (Pref-1)

### 5.1. Brief Overview on the Discovery of Pref-1

In 1987, Helman and colleagues [91,92] investigated genes expressed during differentiation of neuroendocrine tissue. The investigators found two genes, pG2 and pG8, that were highly expressed in both normal and neoplastic neuroendocrine tissue, with pG2 expression being limited to the adrenal gland. A year later, Fay and colleagues discovered a novel protein in amniotic fluid, referred to as fetal antigen-1 (FA-1) [93]. In 1993, Laborda and colleagues reported on a novel protein (delta-like; dlk) belonging to the epidermal growth factor-like superfamily, which is expressed in tumors with neuroendocrine features such as neuroblastoma, pheochromocytoma, and certain small cell lung carcinoma cell lines [94]. The expression of the delta-like (dlk) protein in normal tissue was limited to the adrenal gland and placenta [94]. In the same year, Smas and Sul [73] identified Pref-1 as a novel regulator of adipocyte differentiation. The term “Preadipocyte Factor” was assigned based on the observation that the mRNA was highly expressed in the preadipocyte 3T3-L1 cell line, but not in mature adipocytes. Since then, sequence alignments have shown that pG2, FA1, dlk1 and Pref-1 are the product of the same gene, with varied post transcriptional modifications [95,96].

### 5.2. Pref-1 Structural Characteristics

**Figure 5:**
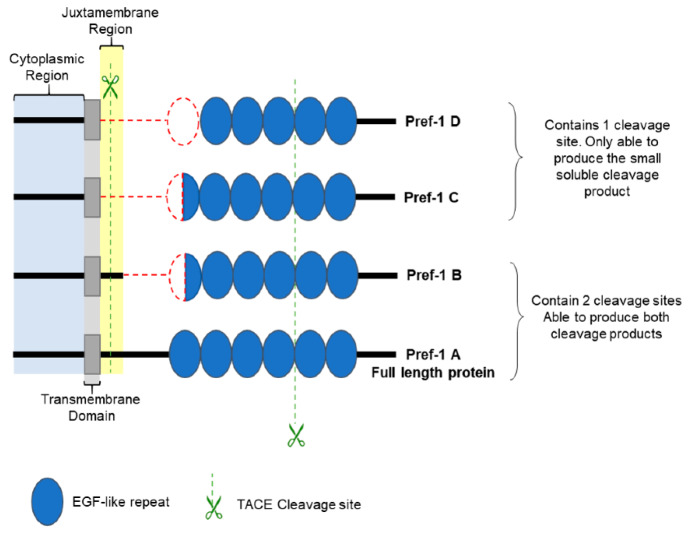
Structure of the different Pref-1 isoforms. The Pref-1 protein can be found in four isoforms (Pref-1 A, B, C and D) due to alternative splicing. The red dotted line indicates the missing sections due to in-frame deletions. Green arrows indicate the cleavage sites for the generation of the soluble forms of the protein. Pref-1, Preadipocyte factor 1; EGF, Epidermal growth factor. Image created by C.d.S.

Pref-1 is a paternally imprinted gene located in an imprinted region of mouse chromosome 12 [97] and on the long arm of chromosome 14 (14q32.2) in humans [71]. The *Pref-1* gene encodes a 385 amino acid protein which exhibits homology to the Notch/Delta/Serrata family of EGF-like proteins [73,74,98,99]. Transmembrane Pref-1 contains an extracellular domain, a juxtamembrane region, a transmembrane domain and a cytoplasmic domain (Figure 5) [74,99,100]. Similar to canonical Notch-ligands, the extracellular domain of Pref-1 contains 6 EGF-repeats (Figure 5), but lacks the N-terminal Delta-Serrate-LAG-2 (DSL) domain found in classical canonical Notch ligands [99,100]. The N-terminal EGF repeats however possess Delta and OSM-11 (DOS) domains common to all Notch ligands [101]. The intracellular domain of Pref-1 is shorter than the classical Notch ligands [102,103]. The Pref-1 protein ranges in size between 50 and 60 kDa due to post translational modifications, such as N-linked glycosylation [99].

Pref-1 has two tumor necrosis factor alpha converting enzyme (TACE) cleavage sites within its extracellular domain, one in the juxtamembrane region and one near the fourth EGF-like repeat (Figure 5). Pref-1 cleavage is regulated by protein kinase C [100]. Cleavage at the juxtamembrane region results in a full-length soluble fraction. Cleavage of the full length soluble fraction at the second cleavage site near the fourth EGF-like repeat produces a large 50 kDa soluble protein fragment as well as a smaller soluble 25 kDa fragment [104,105]. Only the 50 kDa soluble form of the cleaved protein has been implicated as an inhibitor of adipogenesis [74,100,105,106]. The function of the small soluble unit is currently unknown.

Alternative splicing gives rise to four Pref-1 isoforms, Pref-1 A–D (Figure 5) [98]. The Pref-1A isoform is the full-length protein capable of producing both soluble products upon cleavage. Isoforms B to D contain in-frame deletions in the extracellular domain. Nevertheless, despite these deletions, Pref-1 B is capable of producing both soluble forms, as the deletion does not affect either cleavage site (Figure 5). Pref-1 C and D are only capable of producing the small soluble fragment, as the deletion removes the cleavage site within the juxtamembrane region (Figure 5) [74,99].

### 5.3. The Role of Pref-1 in Adipogenesis

The 50 kDa soluble Pref-1 fraction acts in an autocrine/paracrine fashion to regulate both BAT and WAT differentiation [100]. The effect of Pref-1 on adipocyte differentiation was predominantly studied in a murine 3T3-L1 preadipocyte cell line by either over-expressing Pref-1 [73,97,104,107,108,109], by exposure to soluble Pref-1 [106,108,110] or by knocking down *Pref-1* gene expression using Pref-1 anti-sense sequences [72,73,107,108,111]. Over-expression of Pref-1 resulted in impaired adipogenic differentiation, while increased adipogenic differentiation was observed when the *Pref-1* gene was knocked down. The effect of Pref-1 on adipogenic differentiation seems to be cell-type specific, since Nueda and colleagues (2007) found that over-expression of *dlk1* in C3H10T1/2 cells, a murine mesenchymal cell line, potentiates adipogenic differentiation [109]. Similar experimental strategies were employed in vivo using animal models. The expression of adipogenic differentiation-associated genes increased and adipogenic differentiation was accelerated in Pref-1 null mice [71,97], while transgenic mice that over-expressed Pref-1 showed decreased expression of adipogenic differentiation markers, as well as adipose tissue mass [110,112,113].

The role of Pref-1 in BAT differentiation is less well studied. Armengol and colleagues (2012) reported high levels of Pref-1 expression in fetal BAT, which gradually declined after birth [114]. Although these investigators did not observe changes in fetal BAT development in Pref-1 null mice, they noticed that genes specific to brown fat thermogenesis were over-expressed [114]. Furthermore, high levels of Pref-1 expression were observed in C/EBPα-null mice, an experimental model of impaired fetal BAT differentiation, as well as in vitro in primary mouse preadipocytes [114]. Over-expression of Pref-1 in C/EBPα-null mice and primary mouse preadipocytes was accompanied by decreased expression of thermogenic markers such as UCP1 and PPARγ co-activator-α (PGC-1α) [114]. Armengol and colleagues (2012) concluded that Pref-1 could play an important role in controlling thermogenesis-associated gene expression [114]. Zhang and colleagues (2010) observed that treatment of insulin receptor substrate 1 (IRS-1)-deficient brown preadipocytes with bone morphogenetic protein-7 (BMP7) resulted in a significant decrease in Pref-1 expression, which resulted in initiation of adipogenic differentiation. IRS-1-deficient brown preadipocytes express high basal levels of Pref-1 [115]. In a recent study, Rhee and colleagues (2019) investigated the role of Pref-1 in browning of white adipose tissue [116]. Their findings support previous findings suggesting that Pref-1 plays a role in controlling thermogenesis, and they proposed that TACE-mediated cleavage of Pref-1 regulates adipose tissue browning [116].

#### Pref-1 Expression during hASC and hWJSC Adipogenic Differentiation

The role of Pref-1 in adipogenic differentiation has mainly been investigated using the murine 3T3-L1 preadipocyte cell line, which expresses high levels of endogenous Pref-1 [72,73]. Endogenous expression of Pref-1 in hASCs and hWJSCs has not been well studied. Zhang and colleagues (2011) observed Pref-1 (Dlk1) expression in 7 of 15 primary hWJSC cultures [117]. There was no correlation between Pref-1 expression and the ability of the cultures to undergo adipogenic differentiation. However, these investigators did find that the hWJSC cultures that displayed poor adipogenic differentiation did not express Pref-1 [117]. Karagianni and colleagues (2013) found that only a small sub-population of the cells present in primary hWJSC cultures expressed Pref-1 [118]. Furthermore, *Pref-1* gene expression varied greatly between different primary hWJSC cultures. These investigators did not observe significant changes in *Pref-1* expression over a 21-day adipogenic differentiation period, and concluded that the impaired adipogenic differentiation observed in vitro was unrelated to endogenous Pref-1 [118]. Interestingly, high levels of Pref-1 have been observed in umbilical cord blood plasma when compared to maternal plasma [118,119]. Lee and colleagues (2015) were unable to detect Pref-1 in hWJSCs [120], but observed that MSC-like cells isolated from umbilical cord blood expressed high levels of Pref-1 [120,121].

Mitterberger and colleagues (2012) found that the majority of hASCs express Pref-1 and that the expression of Pref-1 decreases during adipogenic differentiation [122]. These investigators also reported that knockdown of the *pref-1* gene resulted in enhanced adipogenic differentiation [122]. To our knowledge this is the only study that has reported on expression of endogenous Pref-1 in hASCs. Zwierzina and colleagues (2015) indicated that approximately 30% of stromal vascular fraction (SVF) cells, isolated from subcutaneous adipose tissue, express cell surface Pref-1 [123]. All SVF cells stained positive for intracellular Pref-1. However, only the cells that were csPref1^Neg^/csCD34^Pos^ (cs, cell surface) displayed strong proliferation and adipogenic differentiation [123].

**Figure 6:**
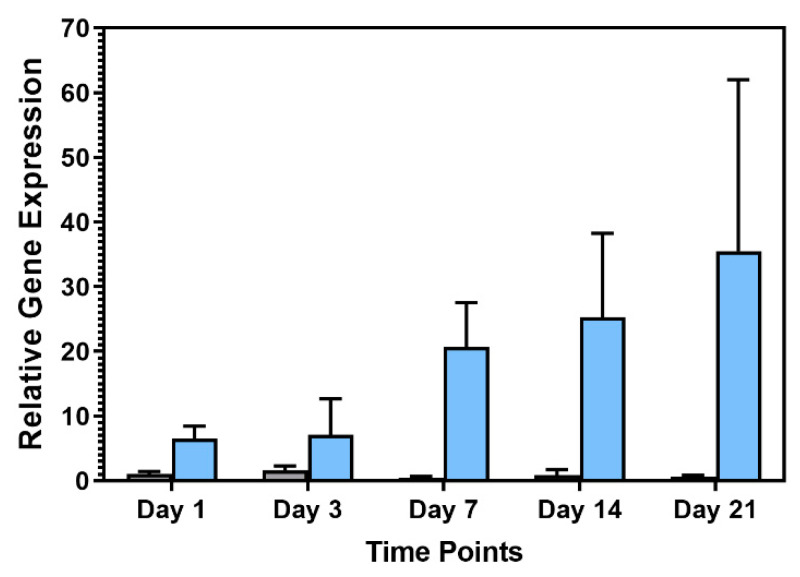
Relative levels of *Pref-1* mRNA expression in hASCs (grey bars) and hWJSCs (blue bars). Data is shown as *Pref-1* expression relative to not induced cells at each time point (*n* = 6 independent isolations for all time points with exception to day 3 where *n* = 3). Gene expression levels were normalized to the following reference genes: *PPIA, TBP, YWHAZ* (ΔCt), after which they were normalized to control (not induced) cells (ΔΔCt). Error bars represent the standard error of the mean (SEM). Unpublished, original data.

Given the differences observed in the adipogenic differentiation potential of hASCS and hWJSCs, and the importance of Pref-1 during adipogenesis, our laboratory investigated *Pref-1* gene expression in primary hASC and hWJSC cultures by reverse transcription-quantitative polymerase chain reaction (RT-qPCR) over a 21-day period. Similar to findings reported by Karagianni and colleagues (2013) [118], we found higher levels of *Pref-1* gene expression in hWJSCs relative to hASCs (Figure 6). Karagianni and colleagues (2013) investigated *Pref-1* gene expression at various time points during adipogenic differentiation of umbilical cord (UC)-derived MSCs, BM-MSCs and ASCs [118]. These authors observed no differences in *Pref-1* gene expression levels during adipogenic differentiation of UC-MSCs and BM-MSCs [118]. Morganstein and colleagues (2010) monitored relative Pref-1 mRNA levels at several time points during adipogenic differentiation of MSCs isolated from fetal blood and fetal bone marrow [124]. Both MSC types (fetal blood and fetal BM) displayed an increase in *Pref-1* gene expression during adipogenic differentiation (compared to the baseline *Pref-1* gene expression levels observed for the respective undifferentiated cells) [124]. Up-regulation of *Pref-1* gene expression was noticeably lower in the fetal blood-derived MSCs when compared to levels observed during adipogenic differentiation of fetal BM-derived MSCs. A gradual increase in *Pref-1* expression was observed until day 14 of adipogenic differentiation for both the fetal blood-derived MSCs and the fetal BM-derived MSCs, after which *Pref-1* gene expression levels decreased [124]. The authors of this review are not aware of any other studies that have investigated the role of Pref-1 during of adipogenic differentiation of MSCs isolated from adipose tissue, UC, blood and BM.

## 6. Pref-1 Mechanism of Action

It is well established that sustained over-expression of Pref-1 prevents adipogenic differentiation. Therefore, Pref-1, needs to be down-regulated for adipogenic differentiation to occur [74,99,100]. However, the exact mechanism by which Pref-1 inhibits adipogenesis has yet to be elucidated. Two pathways have been implicated in Pref-1’s mechanism of action, the mitogen activated protein kinases (MAPK) signaling pathway and the Notch signaling pathway.

### 6.1. Pref-1 and the MAPK Kinase (MEK)/ERK Signaling Pathway

Mitogen-activated protein kinases (MAPKs) play an important role in cell differentiation, proliferation and death by transmitting extracellular signals received by cell surface receptors to transcription factors within the nucleus in order to regulate transcription [125,126]. MAPKs are serine/threonine kinases which can be grouped into three main sub-families: extracellular signal-regulated kinases (ERKs), Jun amino-terminal kinases (JNKs) and stress-activated kinases (p38/SAPKs). The activity of these kinases is regulated by phosphorylation cascades [125,126,127].

Adipocyte differentiation involves cellular growth, proliferation and differentiation, all of which are regulated by MAPKs. It is thus not surprising that MAPK-dependent pathways, especially pathways involving ERK1/2, are involved in adipogenesis. Binding of insulin or IGF-1 to cell surface receptors such as the insulin growth factor receptor, activates the ERK1/2 pathway and promotes adipogenic differentiation [128,129,130]. Insulin-induced adipogenic differentiation is reported to be concentration dependent [131]. Although the exact role of MAPKs in adipogenesis is not fully understood, it is hypothesized that initiation of adipogenic differentiation (mitotic clonal expansion) requires the rapid initial activation of the ERK1/2 pathway [125]. This hypothesis is supported by several studies that have confirmed that initiation of adipogenic differentiation requires early activation of the ERK1/2 pathway [125,132,133,134]. Tang and colleagues (2003) [65] and Kim and colleagues (2007) [129] showed that activation (phosphorylation) of MAPKs occurs within hours of induction of adipogenic differentiation, after which MAPK activity gradually returned to basal levels. Sustained ERK1/2 activation mediates phosphorylation of the master regulator PPARγ, which consequently results in decreased transcriptional activity and adipogenic differentiation [65,128,135]. Down-regulation of ERK1/2 activation is thus needed for adipogenic differentiation to proceed [65,136]. The presence of ERK inhibitors such as PD98059 or UO126 reverses the negative impact of ERK phosphorylation (activation) on adipogenic differentiation [65,136,137].

Using Pref-1 null mouse embryo fibroblasts (MEFs), Kim and colleagues (2007) showed that Pref-1 directly activates the MAPK/ERK (MEK)1/2 and ERK1/2 pathways [129]. An initial, rapid activation (phosphorylation) of ERK1/2, was observed during differentiation of wildtype MEFs into adipocytes. This was followed by a second increase, although lower in intensity, in ERK phosphorylation. The second wave of ERK activation corresponded to Pref-1 expression levels in the wildtype MEFs, but (the second wave) was absent in Pref-1 null MEFs. Enhanced adipocyte differentiation was observed for Pref-1 null MEFs compared to wildtype MEFs. The introduction (supplementation) of soluble Pref-1 during adipogenic differentiation of Pref-1 null MEFs resulted in the restoration of the second wave of ERK activation, which resulted in decreased C/EBPα and PPARγ2 expression and consequently decreased adipogenic differentiation [129]. Nueda and colleagues (2007) observed a similar pattern of ERK activation (initial high level activation followed by a second wave of lower ERK activation) during adipogenic differentiation in C3H10T1/2 cells transfected to express high levels of Pref-1 [109]. The exact manner in which Pref-1 mediates ERK1/2 phosphorylation has, however, not been fully elucidated.

Wang and colleagues (2010) reported that Sox9 is not only involved in chondrocyte and osteogenic differentiation, but also potentially plays a role in adipogenic differentiation [97]. Sox9 is activated by the MEK/ERK pathway [99] and Wang and colleagues (2010) observed that Pref-1-mediated ERK phosphorylation precedes the induction of Sox9 [97]. Activation of Sox9 prevents up-regulation of C/EBPβ and C/EBPδ, which in turn prevents the activation of C/EBPα and PPARγ [97], and ultimately suppresses adipogenic differentiation [99,129,138].

**Figure 7:**
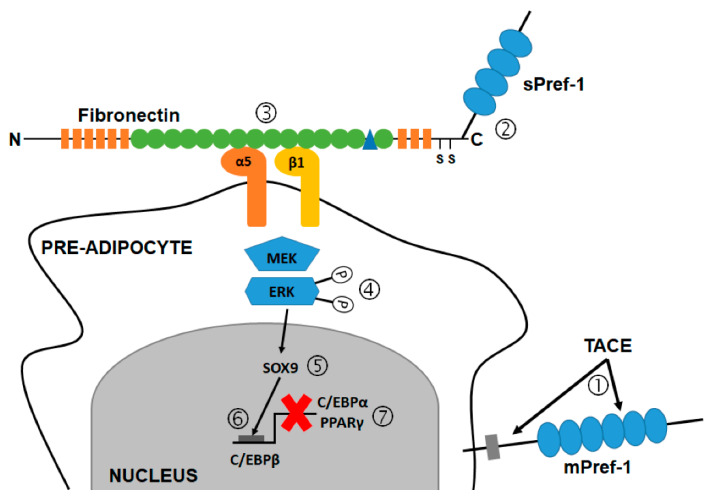
Schematic illustration of the suggested mechanism of Pref-1-mediated inhibition of adipogenesis involving the MEK/ERK pathway. (1) TACE-mediated cleavage of membrane bound Pref-1. (2) 50 kDA soluble Pref-1 binds to the C-terminus of fibronectin. (3) Fibronectin binds to the α5β1 integrin receptor and the fibronectin/sPref-1 complex (4) activates the MEK/ERK pathway, which in turn (5) activates SOX9. (6) SOX9 binds to the C/EBPβ promoter, preventing up-regulation of C/EBPβ and (7) consequently up-regulation of C/EBPα and PPARγ. Figure adapted by C.D. from Hudak and Sul (2013) [100]. C/EBPα, CAAT-enhancer binding protein alpha; C/EBPβ, CAAT-enhancer binding protein beta; ERK, Extracellular signal-regulated kinases; MEK, Mitogen-activated protein kinase kinase; mPref-1, membrane-bound preadipocyte factor-1; sPref-1, soluble preadipocyte factor-1; PPARγ, Peroxisome proliferator-activated receptor gamma; TACE, Tumor necrosis factor alpha converting enzyme.

Fibronectin is a ubiquitous and essential component of the ECM, and plays a role in regulating cellular processes such as cell adhesion, migration, proliferation and differentiation [97,139,140]. Fibronectin is a ligand for many integrin receptor proteins, including the classical fibronectin receptor α5β1 [140]. Wang and colleagues (2010) showed that Pref-1 interacts with the C-terminal region of fibronectin and suggested that the interaction of Pref-1 with fibronectin plays an important role in Pref-1′s inhibition of adipogenesis [97]. Increased adipogenic differentiation was observed when interaction of fibronectin with the α5β1 integrin was disrupted either through treatment with RGD peptides, which compete for binding to α5β1 integrin, or through knock-down of the α5 integrin subunit, or fibronectin, using small interfering RNA (siRNA) transfection [97]. Exposing the cells in which either the α5 integrin subunit or fibronectin was knocked-down, to soluble Pref-1, did not result in a significant decrease in adipogenic differentiation. Wang and colleagues (2010) concluded that the 50 kDA soluble Pref-1 fraction binds to the C-terminal of fibronectin, and thereby facilitates the interaction of Pref-1 with preadipocytes resulting in the activation of the MEK/ERK pathway, which in turn activates Sox9, and thereby inhibits adipogenesis [97,100]. The current suggested mechanism of Pref-1 action is summarized in Figure 7.

There is however currently no consensus regarding the above-mentioned mechanism, for although binding of fibronectin to the α5β1 integrin appears to be important for adipogenesis to occur, how this is impacted by the binding of Pref-1 to fibronectin remains unclear. Zhang and colleagues (2003) suggested that Pref-1 expression impairs ERK1/2 phosphorylation through the down-regulation of IGF-1 [131]. Ruiz-Hidalgo and colleagues (2002) did not observe an increase in ERK1/2 phosphorylation upon induction of Pref-1 expression [141]. Nueda and colleagues (2007) showed that Pref-1 can have both pro- and anti-adipogenic effects, depending on the cell line used [109]: C3H10T1/2 cells displayed poor adipogenic differentiation compared to 3T3-L1 cells. However, the adipogenic capacity of C3H10T1/2 cells increased when Pref-1 was up-regulated through transfection with Pref-1 cDNA constructs encoding the extracellular portion of the protein, while down-regulation of Pref-1 had no effect on these cells. When the same experiments were repeated using 3T3-L1 cells, Pref-1 over-expression inhibited adipogenesis and down-regulation enhanced adipogenesis [109].

### 6.2. Pref-1 and the Notch Signaling Pathway

The Notch signaling pathway present in most animals is highly conserved and is involved in regulating cell fate, proliferation, differentiation and death [142]. Notch refers to a cell-surface receptor that interacts with Notch ligands expressed by surrounding cells [142]. There are five canonical Notch ligands: three Delta-like ligands (DLL1, DLL3, DLL4) and two Jagged ligands (Jagged 1 and 2). The extracellular domain of canonical Notch ligands contains a series of EGF-repeats, a Delta-Serrate-LAG-2 (DSL) domain and an N-terminal domain [143]. The active intracellular domain of the Notch receptor is cleaved by a γ-secretase during Notch ligand/receptor interactions, and relocates to the nucleus where it affects the expression of several transcription factors, such as *Hes1* and *Hey-1* [102,142,144,145].

Initial studies investigated the effect of Pref-1 on Notch signaling in *Drosophilia melanogaster* [103] and *Candida elegans* [146]. Bray and colleagues (2008) reported that membrane-bound Pref-1 was able to antagonize Notch signaling, while soluble Pref-1 had no effect [103]. These findings were contradicted by Komatsu and colleagues (2008), who observed activation of Notch signaling by soluble Pref-1 in *Candida elegans* [146].

Several studies suggest that Notch signaling is involved in the regulation of proliferation and adipogenic differentiation of both white and brown adipocyte progenitor cells [147]. However, the reported findings are often at odds with one another, with some suggesting that Notch signaling does not play an essential role [97,148], potentiates [145,149,150,151,152,153,154] or inhibits adipogenic differentiation [153,155,156,157,158,159,160]. Studies that have reported on the effects of Notch signaling on Pref-1 expression and adipogenic differentiation are summarized in Table 2. It is clear from the differences observed that carefully designed studies, preferably conducted in vivo, are needed to clarify the role of Notch signaling in this context.

Ross and colleagues (2004) indicated that Notch signaling can either potentiate or inhibit 3T3-L1 preadipocyte differentiation [152]. Jagged1-mediated activation of the Notch signaling pathway, as well as over-expression of *hes1*, resulted in decreased C/EBPα and PPARγ expression, and consequently decreased adipogenic differentiation [152]. However, the investigators reported in the same study that knock-down of *hes1* resulted in up-regulation of *Pref-1*, resulting in inhibition of adipogenesis [152]. The investigators attributed their contradictory findings to Hes-1 having a dual role by promoting adipogenic differentiation through Hes-1-mediated down-regulation of inhibitory proteins such as Pref-1, followed by inhibition of adipogenic differentiation by preventing up-regulation of C/EBPα and PPARγ, downstream of Pref-1 (target unknown) [152].

Baladron and colleagues (2004) found that addition of soluble Pref-1 fragments, and over-expression of Pref-1 in 3T3-L1 preadipocytes, resulted in decreased Hes-1 expression and consequently decreased endogenous Notch activity [161]. Furthermore, these investigators supported their findings by showing that knock-down of *Pref-1* resulted in increased Hes-1 expression, suggesting activation of Notch signaling [161]. The investigators only investigated the interaction between Hes-1 and Pref-1 and did not report on any end-stage adipogenesis markers.

Lai and colleagues (2013) reported that the over-expression of Notch 4 resulted in increased proliferation of 3T3-L1 preadipocytes. Furthermore, over-expression of *Notch 4* enhanced the adipogenic differentiation of 3T3-L1 preadipocytes by down-regulating *Pref-1* and up-regulating *C/EBPβ* and *PPARγ* [149].

Nueda and colleagues (2007) investigated the effect of Notch signaling in two different cell lines, 3T3-L1 preadipocytes and mesenchymal C3H10T1/2 cells, which differ with respect to their endogenous Notch1 levels [109]. Garces and colleagues (1997) showed that down-regulation of *Notch1*, using a *Notch1* antisense cDNA construct, prevented adipogenic differentiation in 3T3-L1 cells [151]. Use of the same strategy to knock-down *Notch1* in C3H10T1/2 cells, resulted in decreased adipogenic differentiation [109]. Expression of Pref-1 was unable to overcome the negative effect of decreased Notch signaling on adipogenic differentiation, as it was unable to restore adipogenic differentiation of the *Notch1*-knock-down cells. The investigators concluded that a certain level of Notch1 expression is necessary for cells to undergo adipogenic differentiation [109].

Using primary mouse ASCs (mASCs), Huang and colleagues (2010) showed that inhibition of Notch signaling by inhibition of γ-secretase activity resulted in the down-regulation of *Pref-1* and up-regulation of *PPARγ*, consequently enhancing adipogenic differentiation [162]. Mi and colleagues (2015) showed that the microRNA (miRNA), miR-139-5p, inhibits adipogenic differentiation of 3T3-L1 preadipocytes by directly targeting the untranslated regions (UTRs) of Notch1, resulting in down-regulation of *Hes-1* and *Hey-1*, and up-regulation of *Pref-1* [154].

Song and colleagues (2015) reported down-regulation of *Notch* gene expression during adipogenic differentiation of human BM-MSCs (hBM-MSCs) [158]. Inhibition of Notch signaling resulted in enhanced adipogenic differentiation in hBM-MSCs [155,158]. Interestingly, Vujovic and colleagues (2007) only observed an effect when adipogenic differentiation was induced using only DXM. No effect was observed when differentiation was induced with the complete adipogenic induction cocktail [155].

## 7. Conclusions

Our current knowledge of adipogenesis stems mainly from studies using either the murine 3T3-L1 preadipocytic cell line or murine experimental animal models. It is clear from these studies that Pref-1 plays an important regulatory role during murine adipocyte differentiation. However, the role of Pref-1 during adipogenic differentiation in humans is not well established. Furthermore, it is unclear if the results from murine studies can be directly translated to the human setting, and more studies using cells of human origin are needed. In this regard, the observed differences in the ability of human MSCs of different origins to undergo adipogenesis in vitro, provides an excellent model to study human adipocyte differentiation.

## Figures and Tables

**Table 1:** Summary of the adipogenic differentiation potential of MSCs isolated from human tissues. The sources are listed from left to right according to decreasing adipogenic potential observed in the respective studies (direct comparisons).

Study	Tissue Sources Compared																	Reference
Amable et al., 2014	AT			>	BM					>	WJSC							[46]
Brohem et al., 2013	AT			>	BM													[49]
Hu et al., 2013	AT									=	WJSC							[47]
Li et al., 2015	AT			=	BM													[50]
Liu et al., 2009	AT			>	BM													[51]
Manini et al., 2011	AT			>	BM	>	Dermis											[52]
Maurney et al., 2007	AT			=	BM													[53]
Mohamed-Ahmed et al., 2018	AT			>	BM	>	Dermis											[54]
Noël et al., 2007	AT			=	BM													[55]
Ragni et al., 2013	AT	>			BM	>	UCB			>	WJSC	>	AF	>	PV			[45]
Sakaguchi et al., 2005	AT	=	SV	>	BM	=	PS	>	Muscle									[56]
Xu et al., 2017	AT			>	BM													[30]
Baksh et al., 2007			UC	>	BM													[57]
Barlow et al., 2015					BM							>	PC					[58]
Batsali et al., 2017					BM					>	WJSC							[59]
Isobe et al., 2015					BM							=	SF	>	SHED	>	DP	[60]
Zhu et al., 2012				>	BM													[61]

AF, amniotic fluid; AT, adipose tissue; BM, bone marrow; DP, dental pulp; nFS, neonatal foreskin; SHED, exfoliated deciduous teeth; PS, periosteum; PV, perivascular region of UC; SF, synovium fluid; SV, synovium; PC, placenta; UCB, umbilical cord blood; UC, umbilical cord; =, no difference in adipogenic potential was observed.

**Table 2:** Summary of the impact of Notch signaling on Pref-1 expression and adipogenic differentiation.

Cell Type	Experimental Approach	Impact on			References
		Notch signaling	Adipogenic Differentiation	Pref-1 Expression	
3T3-L1	Exposure to soluble Jagged1	Up-regulated	Decreased	Not determined	[152]
	*hes1* over-expression	Up-regulated	Decreased	Not determined	
	*hes1* knock-down	Down-regulated	Decreased	Up-regulated	
3T3-L1	Notch4 over-expression	Up-regulated	Increased	Down-regulated	[149]
3T3-L1	Adipogenic differentiation. No specific treatment.	Decreases over time	Increased (compared to C3H10T1/2)	Not determined	[109]
	*dlk1* knock-down	Up-regulated	Not assessed	Down-regulated	
C3H10T1/2	Adipogenic differentiation. No specific treatment.	Remained unchanged	Decreased (compared to 3T3-L1 cells)	Up-regulated	
	*dlk1* over-expression	Down-regulated	Increased	Up-regulated	
	*dlk1* knock-down	Up-regulated	Increased	Down-regulated	
	*Notch1* knock-down	Down-regulated	Decreased	Down-regulated	
	*dlk1* over-expression in *Notch1* knock-down cells	Down-regulated	Decreased	Up-regulated	
3T3-L1	Transfection with miRNA (miR-139–5p mimic)	Initially increased. Gradually decreased.	Decreased	Up-regulated	[154]
	Co-transfection with pcDNA3.1_NICD (over-expressing Notch)	Up-regulated	Increased	Not determined	
Mouse BM-derived MSCs	Inhibition of γ-secretase	Decreased	Increased	Decreased	[162]
Human BM-derived MSCs	Adipogenic differentiation. No specific treatment.	Decreased	Increased	Decreased	[158]
	Inhibition of γ-secretase	Decreased	Increased	Not measured	

siRNA, small interfering RNA; miRNA, micro-RNA; BM, bone marrow

## Abbreviations

AF	Amniotic fluid
AP-1	Activating protein-1
aP2	Adipocyte protein 2
ASCs	Adipose-derived stromal/stem cells
AT	Adipose tissue
BAT	Brown adipose tissue
BM	Bone marrow
BM-MSCs	Bone marrow-derived MSCs
BMP7	Bone morphogenetic protein-7
C/EBP	CAAT-enhancer binding proteins
cAMP	Cyclic Adenosine Monophosphate
CHOP	C/EBP homologous protein
cs	Cell surface
DLK	Delta-like
DLL	Delta-like ligands
DOS	Delta and OSM-11
DP	Dental pulp
DSL	Delta-Serrate-LAG-2
DXM	Dexamethasone
ECM	Extracellular matrix
EGF	Epidermal growth factor
ERKs	Extracellular signal-regulated kinases
FA-1	fetal antigen-1
FABP4	Fatty acid-binding protein 4
FBS	Fetal bovine serum
Foxp1	Foxhead P1
hASCs	Human-derived ASCs
hBM-MSCs	Human-derived BM-MSCs
hWJSCs	Human-derived WJSCs
IBMX	3-Isobutyl-1-methylxantine
IGF-1	Insulin-like growth factor 1
IRS-1	Insulin receptor substrate 1
JNKs	Jun amino-terminal kinases
kDa	kilo Dalton
KLFs	Krüppel-like factors
LDL	Low-density lipoprotein
MAPK	Mitogen-activated protein kinase
mASCs	Mouse ASCs
MEFs	Mouse embryo fibroblasts
MEK	Mitogen-activated protein kinase kinase
mPref-1	Membrane bound Pref-1
mRNA	Messenger ribonucleic acid
MSCs	Mesenchymal stromal/stem cells
Myf-5	Myogenic factor 5
nFS	Neonatal foreskin
PC	Placenta
PECK	Phosphoenolpyruvate carboxylase
PGC-1α	PPARγ co-activator-alpha
PPARγ	Peroxisome proliferator-activated receptor gamma
PRDM16	PR domain containing 16
Pref-1	Preadipocyte factor-1
PS	Periosteum
PV	Perivascular region
RT-qPCR	Reverse transcription-quantitative polymerase chain reaction
SAPKs	Stress-activated kinases
SF	Synovium fluid
SHED	Exfoliated deciduous teeth
SMAD	Mothers against decapentaplegic homolog
SOX	Sex determining region Y-box
SV	Synovium
sPref-1	Soluble Pref-1
SREBP-1	Sterol regulatory element binding protein 1
STAT	Signal transducer and activator of transcription
SVF	Stromal vascular fraction
TACE	Tumor necrosis factor alpha converting enzyme
UC	Umbilical cord
UCB	Umbilical cord blood
UCP1	Uncoupling protein 1
UTR	Untranslated region
WAT	White adipose tissue
WJSCs	Wharton’s jelly derived stromal/stem cells
Wnt	Wingless/integrated protein
ZEB1	Zinc finger E-box binding homeobox 1
